# The mGluR5 Antagonist Fenobam Induces Analgesic Conditioned Place Preference in Mice with Spared Nerve Injury

**DOI:** 10.1371/journal.pone.0103524

**Published:** 2014-07-25

**Authors:** Neil C. Lax, David C. George, Christopher Ignatz, Benedict J. Kolber

**Affiliations:** Department of Biological Sciences and Chronic Pain Research Consortium, Duquesne University, Pittsburgh, Pennsylvania, United States of America; Boston Children's Hospital and Harvard Medical School, United States of America

## Abstract

Antagonists of metabotropic glutamate receptors (mGluRs) have the potential to act as analgesic drugs that may help alleviate chronic pain. This study was done to look at the possible rewarding properties of the mGluR5 antagonist, fenobam, in a cognitive assay. Analgesic conditioned place preference (aCPP) was used to examine the effects of fenobam (30 mg/kg) and the prototypical mGluR5 antagonist, MPEP, and these effects were compared to those of a drug with known analgesic properties, morphine (10 mg/kg). In each experiment, one group of mice received spared nerve injury (SNI) surgery to model chronic pain; the other group received a control sham surgery. Both fenobam and MPEP induced preference in the SNI mice, such that SNI mice spent significantly more time in the mGluR5 antagonist-paired chamber compared to a vehicle-paired chamber. No such preference developed for sham mice. Morphine induced preference in male and female mice in both the SNI and sham groups. The results showed that fenobam and MPEP likely reduced on-going distress in the SNI mice, causing them to prefer the chamber paired with the drug compared to the vehicle-paired chamber. Since sham animals did not prefer the drug-paired chamber, these data demonstrate that mGluR5 antagonism is non-rewarding in the absence of pain-like injury.

## Introduction

Over 100 million people in the United States suffer from chronic pain at some point in their lifetimes, making this one of the most widespread of medical conditions [1]. Despite the prevalence of this condition, options are limited for patients seeking treatment. Non-steroidal anti-inflammatory drugs (NSAIDs) and opioid drugs, such as morphine, remain the most commonly prescribed medications for chronic pain sufferers [2]. These drugs, which can have deleterious side effects, often do not work for patients [3]–[5]. Recently, metabotropic glutamate receptor 5 (mGluR5) has emerged as a potential new target in the treatment of chronic pain.

mGluR5 is a G-protein coupled receptor localized to regions of the periphery, spinal cord and brain involved with the processing of pain [6]–[8]. Activation of mGluR5 receptors in the spinal cord and amygdala, using (*R,S*)-3,5-dihydroxyphenylglycine (DHPG), causes pain-like behaviors in mice, while inhibition with the prototypical antagonist, 2-methyl-6-(phenylethynyl)pyridine hydrochloride (MPEP), decreases these responses [9], [10]. This has led to the search for other mGluR5 agents, such as fenobam [N-(3-chlorophenyl)-N′-(4,5-dihydro-1-methyl-4-oxo-1H-imidazole-2-yl)urea], that could be used to target mGluR5. Fenobam was first developed in the 1970s as an anxiolytic drug, but was later found to be a specific noncompetitive antagonist of mGluR5 [11]. Using models of inflammatory pain, such as the Complete Freund's Adjuvant (CFA) and formalin tests, fenobam acts as an effective analgesic-like agent at 30 mg/kg [12]. In animals, fenobam has a good safety profile and there is no evidence for the development of long-term tolerance in mice [13]. However, the action of fenobam has only been demonstrated in classic acute reactive pain tests in which nocifensive responses are directly induced by an experimenter. It remains unclear if fenobam relieves on-going spontaneous chronic pain or affects naïve animals. Additionally, only male mice have been studied in the context of fenobam treatment. It is important to consider the effects of fenobam in both sexes since more women are reported to suffer from chronic pain than men [14] and since there are sex differences in degrees of pain and analgesia [15], [16].

Given the clinical potential of fenobam, we tested if fenobam would induce place preference in the context of neuropathic injury, a chronic pain pathology. We also determined if the effects of fenobam were coupled with the rewarding tendencies in the absence of any discomfort commonly seen in many pain-relieving drugs. Spared Nerve Injury (SNI), a model of spontaneous chronic neuropathic pain [17], was used in conjunction with the analgesic Conditioned Place Preference (aCPP) assay. SNI has been shown repeatedly to induce mechanical hypersensitivity in the affected limb for up to nine weeks post-surgery [17]–[19]. Conditioned place preference has been widely used to test the rewarding and addictive potentials of numerous drugs [20]. A new variant of this assay, aCPP, utilizes positive reinforcement to detect and treat non-evoked spontaneous pain. In the aCPP assay, animals learn to associate a physical visual cue with the effects of a particular drug. Using this assay, rats and mice with Spared Nerve Ligation (SNL) or Complete Freund's Adjuvant (CFA) injury developed a preference for clonidine (mice and rats) [21], omega conotoxin (rats) [21], or lidocaine (mice) [22], drugs known to alleviate pain in humans [23], [24]. In these studies, animals with sham injuries (and therefore no hypersensitivity) showed no place preference. We hypothesized that fenobam, based on its molecular target and known effects, could produce aCPP in mice with SNI. In addition, we compared the fenobam results to results obtained with the mGluR5 antagonist, MPEP, in order to confirm that the effects were due to the antagonism of mGluR5. Subsequently, we wanted to compare the fenobam and MPEP results to results obtained with morphine, which induces analgesia, but which also induces euphoria and addiction in uninjured individuals [25].

## Materials and Methods

### Animals

All mouse procedures were reviewed and approved in accordance with National Institutes of Health Guide for the Care and Use of Animals and the Institutional Animal Care and Use Committee at Duquesne University (Protocol Number: 1201-02). All behavior experiments were performed using C57Bl/6J male or female mice. Mice were individually housed and maintained on a 12-hour light/dark cycle (lights on 7:00 AM–7:00 PM) with *ad libitum* access to food and water. Mice were between 6.5 and 8 weeks old when behavioral experimentation took place. All procedures were carried out during the light cycle. The mouse's surgery type was blinded to the experimenter prior to all behavioral testing.

### Surgical Procedures

A 10∶1 ketamine/xylazine mixture was injected intraperitoneally into the mice for anesthesia (10 µL/g). Spared nerve injury (SNI) to the sciatic nerve was performed as described previously [19]. Briefly, a suture thread was tied around tibial and common peroneal branches of the sciatic nerve, both of which were ligated 2 cm distal to the suture. The sural branch of the sciatic nerve was not manipulated. Sham surgeries followed the same procedure, without manipulation of any branches of the sciatic nerve. Mice recovered on heating pads and were housed in individual cages for one week prior to aCPP testing. Following all behavioral procedures, sham and SNI surgeries were verified *post-hoc* with necropsy.

### Drugs

Fenobam ([N-(3-chlorophenyl)-N′-(4,5-dihydro-1-methyl-4-oxo-1H-imidazole-2-yl)urea], Abcam Biochemicals, Fenobam, Cambridge UK) was dissolved in 100% dimethyl sulfoxide (DMSO) on the first day of drug-pairing (day 2 of 5-day aCPP experiment) at a dose of 30 mg/kg (volume = 20 µL) and stored in the dark at room temperature between tests. Dosage was determined from published dose response curves [12] and our own preliminary data showing significant analgesic effects of fenobam in the spontaneous formalin test (data not shown). DMSO was chosen as the vehicle due to its use with fenobam in other pain and pharmacological studies [12], [13], [26] and fenobam's lack of solubility in other solvents. MPEP ([2-Methyl-6-(phenylethynyl)pyridine], Enzo Life Sciences, MPEP hydrochloride, New York USA) was dissolved in 0.9% saline on the first day of drug-pairing (day 2 of 5-day aCPP experiment) at a dose of 30 mg/kg (volume = 20 µL) and stored in the dark at room temperature between tests. Dosage was determined from published dose response curves [12]. Morphine (Sigma, morphine sulfate, USA) was dissolved in 0.9% saline solution on the first day of drug pairing (day 2 of the 5-day aCPP experiment) at a dose of 10 mg/kg (volume = 100 µL) and stored in the dark at room temperature between tests. The dose for morphine was determined from previous studies showing CPP for morphine in naïve mice [27], [28].

### Drug Administration

Fenobam solution was administered intraperitoneally (30 mg/kg) in a volume of 20 µL, 5 minutes prior to behavioral testing, when fenobam concentration in the brain is maximal [12]. The vehicle control for fenobam trials was 100% DMSO (volume = 20 µL). MPEP solution was administered intraperitoneally (30 mg/kg) in a volume of 20 µL, 5 minutes prior to behavioral testing as well. The vehicle control for MPEP trials was 0.9% saline solution (volume = 20 µL). Morphine was administered to mice subcutaneously (10 mg/kg) in a volume of 100 µL, 5 minutes prior to behavioral testing. The vehicle control for the morphine trials was 0.9% saline (volume = 100 µL). In separate trials of 5-day aCPP tests (see below), fenobam, MPEP, or morphine was given to mice once daily for three consecutive days (on days 2, 3, and 4 of aCPP).

### Analgesic Conditioned Place Preference (aCPP)

The aCPP apparatus consisted of a three-compartment box made from Plexiglas. The box consisted of two large outer chambers (26.75 cm×27.5 cm) and one smaller middle chamber (“neutral” chamber – 9 cm×5 cm) that connected the two outer chambers. The two large outer chambers differed only in the patterns on the wall (vertical, horizontal or diagonal black and white stripes, all 2.675 cm thick), while the rest of the parameters remained identical between chambers (white noise 60 dB, non-enrichment bedding). The neutral chamber in the middle of the apparatus consisted of white walls with no bedding.

On day 1 of aCPP testing, seven days following SNI/sham surgeries, the male or female mice were placed in the neutral chamber and allowed to move freely between the three chambers for 30 minutes. Mice were tracked by two overhead cameras (Logitec Webcam Pro 9000 and Canon ZR420) using ANY-maze software (Stoelting Co., version 4.98). This program recorded the time spent and distance traveled by each mouse in all three chambers on days 1 and 5 of aCPP testing. Additionally, the distance traveled on days 2–4 for male mice treated with fenobam was recorded. Day 1 established baseline results upon which the balancing of the apparatus was assessed. In order to avoid preconditioning biases, we made the *a priori* decision to exclude any mouse that had a difference of greater than 400 seconds in either of the two outer chambers (vertical vs horizontal/diagonal) on day 1 (pre-conditioning) of aCPP testing. For remaining animals that passed the exclusion test, on days 2, 3, and 4, drug or vehicle was administered in two separate 30-minute sessions (one session in the morning and one in the afternoon). During these trials, the neutral chamber was sectioned off and the mice only had access to one of the large outer chambers (either vertical or horizontal/diagonal). In the morning session, mice were injected with the vehicle control and placed in the vehicle-paired outer chamber. Three hours later, in the afternoon trial, the same mice were injected with the experimental drug (i.e. fenobam, MPEP or morphine in vehicle) and placed in the drug-paired outer chamber, opposite to the chamber used in the morning session. The chamber paired with the drug was randomly assigned to mice such that some mice received the drug in a vertical chamber and others in a horizontal/diagonal chamber. On day 5 (post-conditioning), mice were again allowed to roam free between all three chambers for 30 minutes. These new times were then compared to day 1 (pre-conditioning) testing times to see if and how the mouse's preference changed after three days of drug-pairing.

### Statistical Analysis

Graph Pad Prism version 5 for Mac OS X was used for all statistical analysis. All data are presented as mean ± SEM. Raw data can be obtained by contacting the corresponding author. Statistical significance was determined between groups using t-tests or ANOVA followed by a *post ho*c test. The combined total distances were analyzed with an un-paired t-test and days 2–4 distance data were analyzed with a two-way ANOVA followed by a Bonferroni *post-hoc* test. When analyzing total time per chamber on day 5, a paired t-test was utilized. Day 5 – Day 1 time difference data was analyzed using a paired t-test. Statistical significance was established at a 95% confidence interval (p<0.05).

## Results

### aCPP with fenobam in male mice

**Figure 1:**
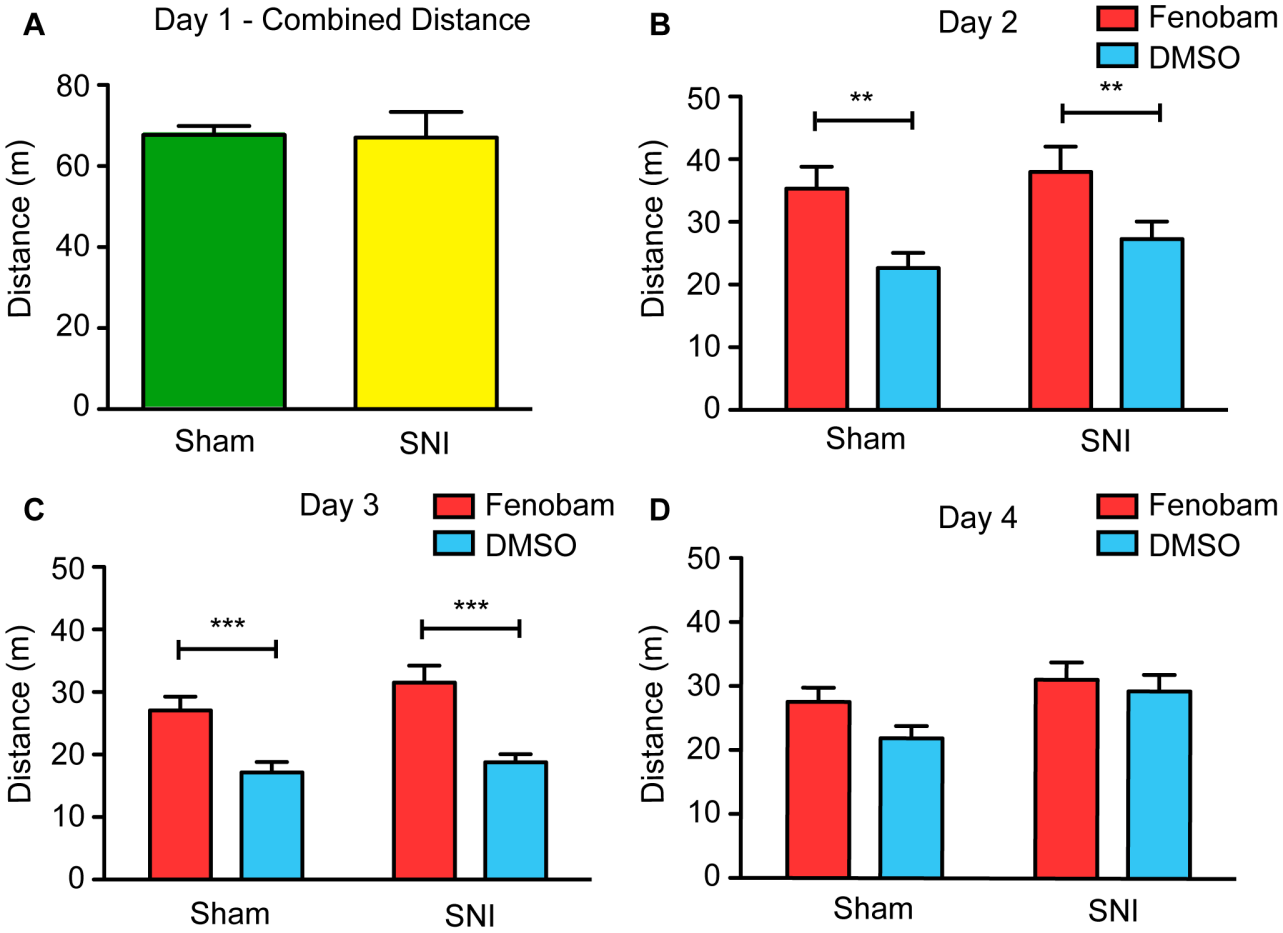
Male SNI and sham mice travel similar distances and show tolerance to locomotor side-effects. (**A**) SNI (n = 15) and sham (n = 15) mice travel a similar distance in both outer chambers of the aCPP apparatus on preconditioning day 1. (**B**) On conditioning day 2 and (**C**) conditioning day 3, both SNI and sham mice are hyperactive when treated with fenobam. Mice were restricted to only one of the outer chambers when these measurements were taken. (**D**) However, by day 4, neither group of mice show increased distance traveled in the fenobam chamber compared to the vehicle-paired chamber. Two-way ANOVA followed by Bonferroni *post hoc* test, **p<0.01, ***p<0.001.

Using the aCPP assay, we first evaluated the analgesic effect of fenobam (30 mg/kg) in male mice with SNI surgery (n = 15) compared to male mice with a control sham surgery (n = 15). Baseline data for the time and distance traveled during 30 minute trials in the aCPP apparatus was collected on preconditioning day 1. SNI injury is associated with behavioral hypersensitivity [17] and impaired movement due to loss of sensory and motor neurons, respectively. Thus, we compared the total distance traveled by sham and SNI animals on pre-conditioning day 1 in an attempt to see if either group showed a reduction in locomotor output. Comparing the overall distance traveled in both of the large outer chambers, we failed to find a statistically significant difference between SNI and sham operated mice (Figure 1A; un-paired t-test, t_(28)_ = 0.096, p = 0.9246).

Following day 1, vehicle and fenobam were paired with their own respective chambers on conditioning days 2–4. During this time, the mice were conditioned to associate the effects of the vehicle control with one of the outer chambers and the effects of fenobam with the other outer chamber. On each of the conditioning days, the total distance traveled in the vehicle-paired chamber was recorded in the morning, and the total distance traveled in the fenobam-paired chamber was recorded in the afternoon. We found that both SNI and sham groups traveled significantly more after administration of the fenobam compared to administration of the vehicle on day 2 (Figure 1B; 2-way ANOVA, significant main effect of treatment, F_(1,29)_ = 22.1, Bonferroni *post-hoc* test, p<0.01) and day 3 (Figure 1C; 2-way ANOVA, significant main effect of treatment, F_(1,29)_ = 43.80, Bonferroni *post-hoc* test, p<0.001). However, no statistically significant hypermobility was found on day 4 in either group of mice (Figure 1D; 2-way ANOVA, no significant main effect of treatment, F_(1,29)_ = 2.856, p>0.05).

**Figure 2:**
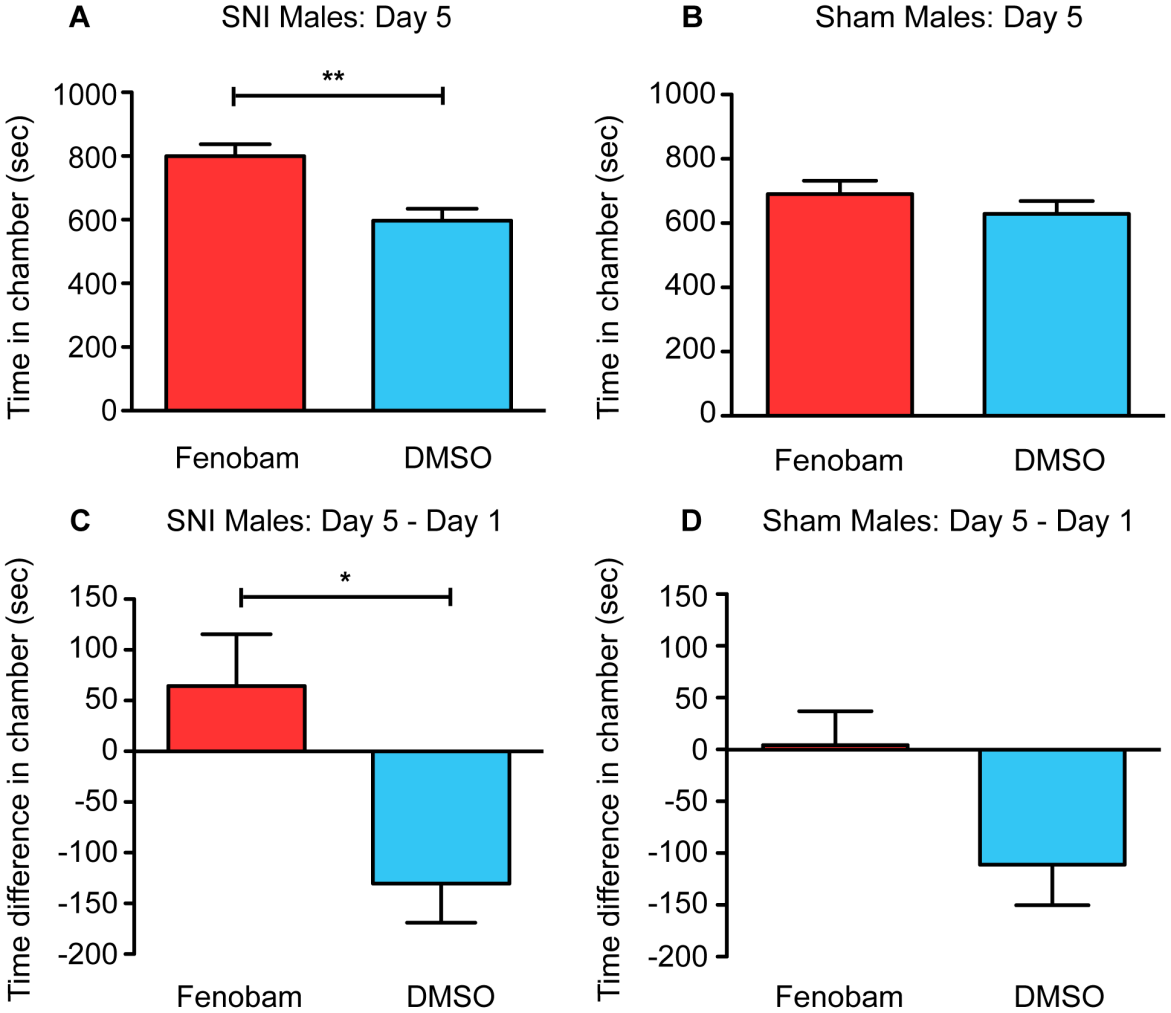
Fenobam induces place preference in male SNI mice only. (**A**) SNI mice (n = 15) spent significantly more total time in the fenobam-paired chamber compared to vehicle-paired chamber on post-conditioning day 5. (**B**) Sham mice (n = 15) show no difference in the total amount of time spent in fenobam and vehicle-paired chamber on post-conditioning day 5. (**C**) SNI mice increase the amount of time they spent in the fenobam-paired chamber on day 5 (post-conditioning) compared to baseline (day 1 pre-conditioning) while (**D**) sham mice show no such difference. Paired t-test, *p<0.05, **p<0.01.

On post-conditioning day 5, mice were exposed again to the entire apparatus (i.e. all three chambers) for 30 minutes. By comparing the times spent in each chamber on day 5 to day 1, it was possible to see if preferences for the chambers changed after the three days of pairing. We used two methods of analysis to look for preference for the fenobam chamber in SNI or sham mice. First, we directly compared the time spent in the fenobam-paired chamber on day 5 to the time spent in the DMSO-paired chamber on day 5. Indicative of a preference for fenobam, SNI mice spent more time in the fenobam-paired chamber compared to the DMSO-paired chamber on day 5 (Figure 2A; paired t-test, t_(14)_ = 3.07, p = 0.0084). In contrast, sham mice did not show an increase in time spent in the fenobam chamber compared to the DMSO-paired chamber on day 5 (Figure 2B; paired t-test, t_(14)_ = 0.85, p = 0.409). Second, we accounted for small individual baseline biases by calculating the difference in time spent in each chamber (e.g. fenobam-paired chamber, vehicle-paired chamber) on day 1 compared to day 5. We found that SNI mice spent more time in the fenobam-paired chamber (day 5 minus day 1) than the DMSO-paired chamber (day 5 minus day 1) (Figure 2C, paired t-test, t_(14)_ = 2.37, p = 0.033). In contrast, sham-operated mice did not show a significant difference between the time spent in the fenobam-paired chamber, compared to the DMSO-paired chamber (Figure 2D; paired t-test, t_(14)_ = 1.78, p = 0.097).

### aCPP with fenobam in female mice

**Figure 3:**
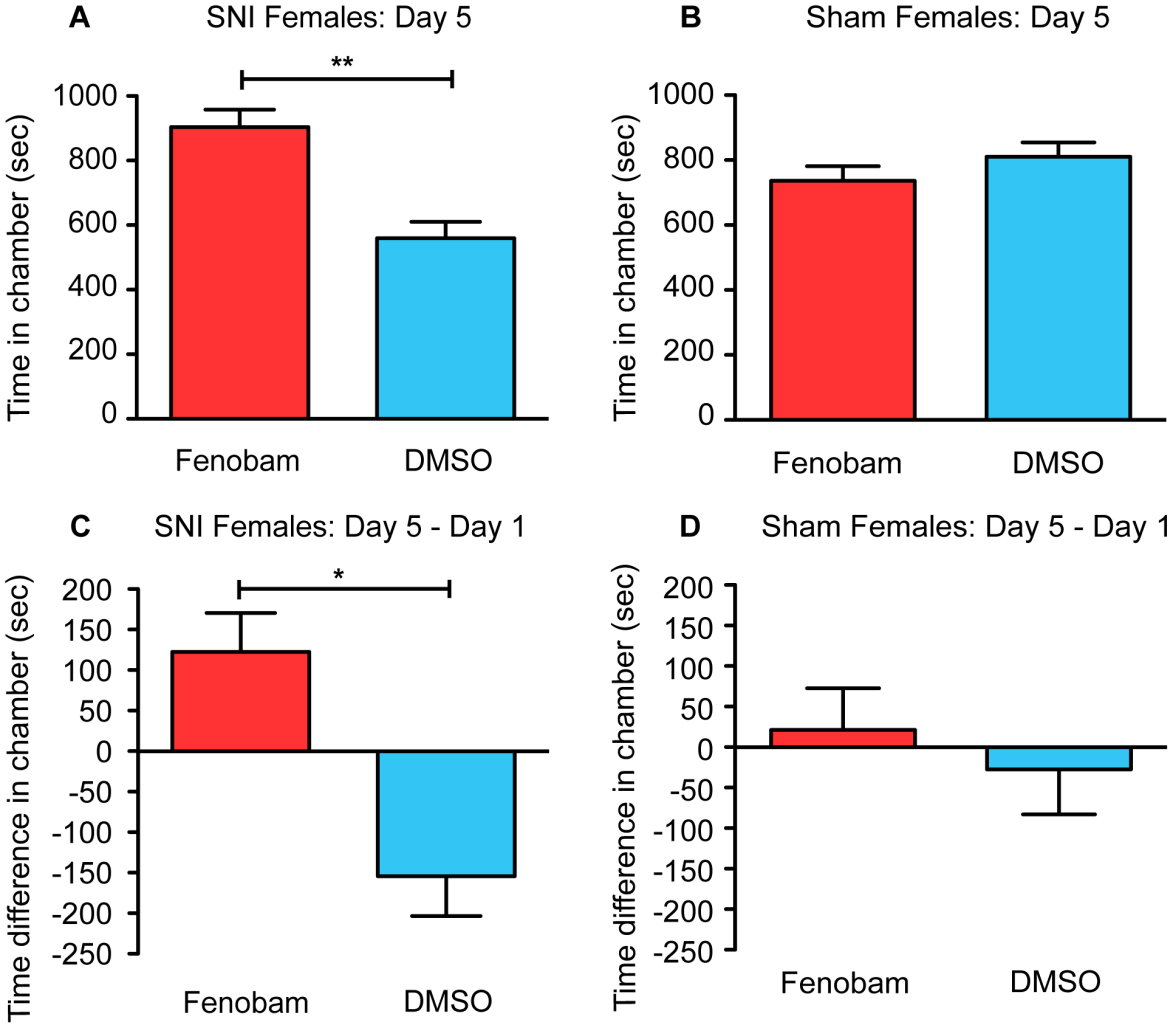
Fenobam induces place preference in female SNI mice only. (**A**) SNI mice (n = 10) spent significantly more total time in the fenobam-paired chamber compared to vehicle-paired chamber on post-conditioning day 5. (**B**) Sham mice (n = 11) show no difference in the total amount of time spent in fenobam and vehicle-paired chamber on post-conditioning day 5. (**C**) SNI mice increase the amount of time they spend in the fenobam-paired chamber on day 5 (post-conditioning) compared to baseline (day 1 pre-conditioning) while (**D**) sham mice show no such difference. Paired t-test, *p<0.05, **p<0.01.

Since there are significant reported gender differences in chronic pain conditions and treatment [14]–[16], we next tested if fenobam would induce similar conditioning in female SNI mice. We found no difference between SNI and sham mice in the total distance traveled on day 1 (Sham mice = 79.60±6.617 m, SNI mice = 69.52±7.135 m, un-paired t-test, t_(19)_ = 1.04, p = 0.312). Analysis of preference data on day 5 showed similar results to those seen in male mice; female SNI mice (n = 10) spent more time in the fenobam-paired chamber than the DMSO-paired chamber on day 5 (Figure 3A; paired t-test, t_(9)_ = 3.54, p = 0.006). For the sham-treated female mice (n = 11), there was no preference for the fenobam-paired chamber over the DMSO-paired chamber on day 5 (Figure 3B; paired t-test, t_(10)_ = 0.86, p = 0.409). When day 5 data was compared to day 1, we saw that SNI mice spent more time (day 5 minus day 1) in the fenobam-paired chamber than the DMSO-paired chamber (Figure 3C; paired t-test, t_(9)_ = 3.15, p = 0.012). Conversely, sham treated mice showed no significant increase in the time spent in the fenobam-paired chamber when compared to the DMSO-paired chamber (Figure 3D; paired t-test, t_(10)_ = 0.46, p = 0.656).

### aCPP with MPEP in male mice

**Figure 4:**
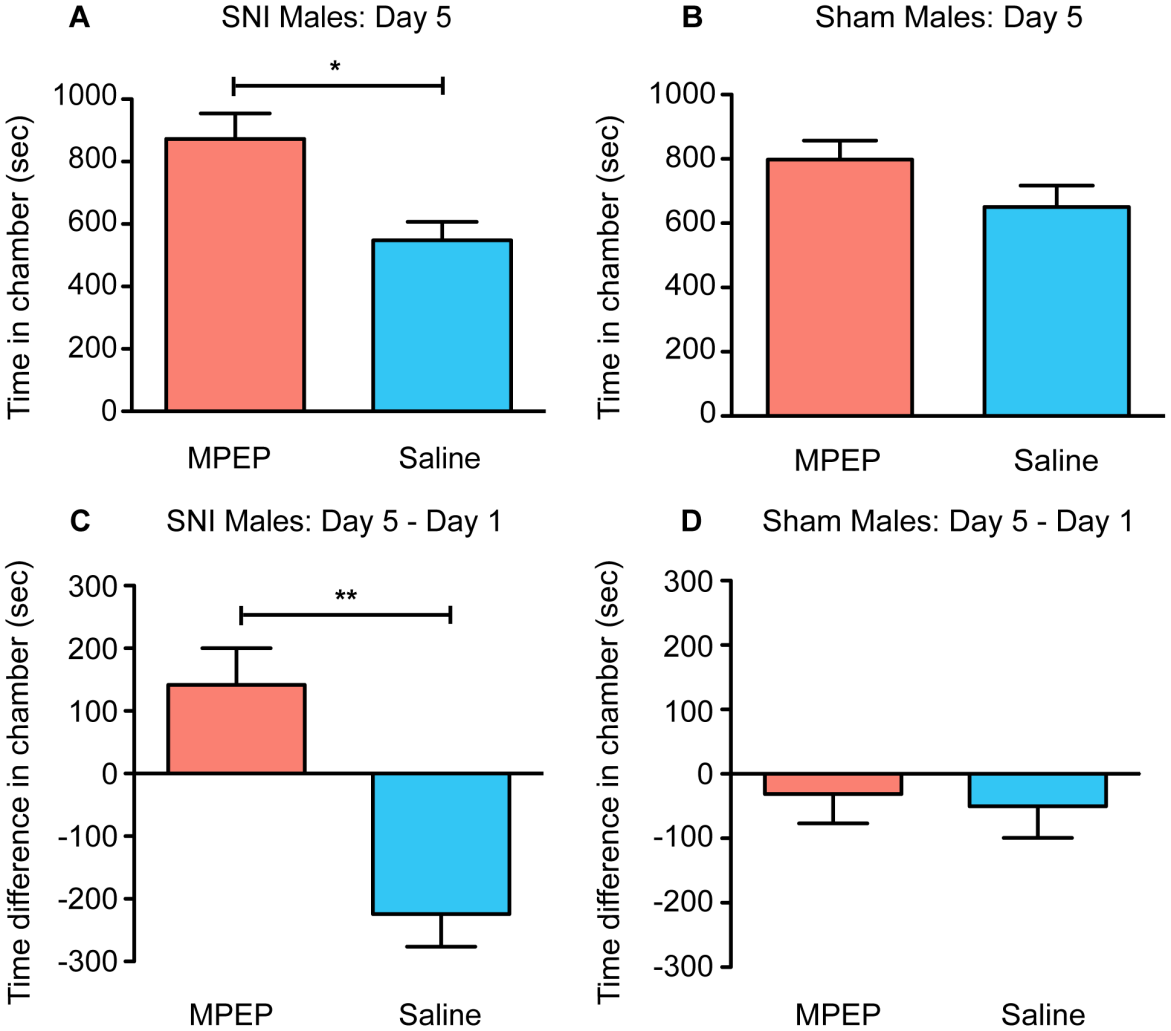
MPEP induces place preference in male SNI mice only. (**A**) SNI mice (n = 8) spent significantly more total time in the MPEP-paired chamber compared to vehicle-paired chamber on post-conditioning day 5. (**B**) Sham mice (n = 8) show no difference in the total amount of time spent in MPEP and vehicle-paired chamber on post-conditioning day 5. (**C**) SNI mice increase the amount of time they spend in the MPEP-paired chamber on day 5 (post-conditioning) compared to baseline (day 1 pre-conditioning) while (**D**) sham mice show no such difference. Paired t-test, *p<0.05, **p<0.01.

In order to confirm that the preference induced by fenobam in the SNI mice was due to antagonism of mGluR5, an additional aCPP assay was performed using the prototypical mGluR5 antagonist MPEP. We hypothesized that the same preference would be induced in the SNI mice, while not affecting the sham control mice. In this experiment, the same aCPP assay was used, but MPEP (30 mg/kg) and saline (0.9%) were substituted for fenobam and DMSO respectively on days 2–4 of testing. Unlike fenobam, MPEP can be dissolved in saline solution. Analysis of the preference data on day 5 replicated the aCPP results that were obtained with fenobam; male SNI mice (n = 8) spent more time in the MPEP-paired chamber than the saline-paired chamber on day 5 (Figure 4A; paired t-test, t_(7)_ = 2.566, p = 0.0372). For the sham mice (n = 8), no preference developed for the MPEP-paired chamber over the saline-paired chamber on day 5 (Figure 4B; paired t-test, t_(7)_ = 1.301, p = 0.2344). In the same manner that was seen with the fenobam data, when day 5 data was compared to day 1, we saw that SNI mice spent more time (day 5 minus day 1) in the MPEP-paired chamber than the saline-paired chamber (Figure 4C; paired t-test, t_(7)_ = 3.828, p = 0.0065). Sham mice did not increase the time that they spent in the MPEP-paired chamber when compared to the saline-paired chamber (Figure 4D, paired t-test, t_(7)_ = 0.2507, p = 0.8092).

### aCPP with morphine in male mice

Having demonstrated that fenobam induced preference in male and female mice with SNI but not in sham-operated mice, we next examined the effect of morphine in the aCPP assay, hypothesizing that all mice, regardless of surgery type, would show conditioned place preference for the drug. The same aCPP assay was used, but morphine (10 mg/kg) and saline (0.9%) were used on days 2–4 of testing.

**Figure 5:**
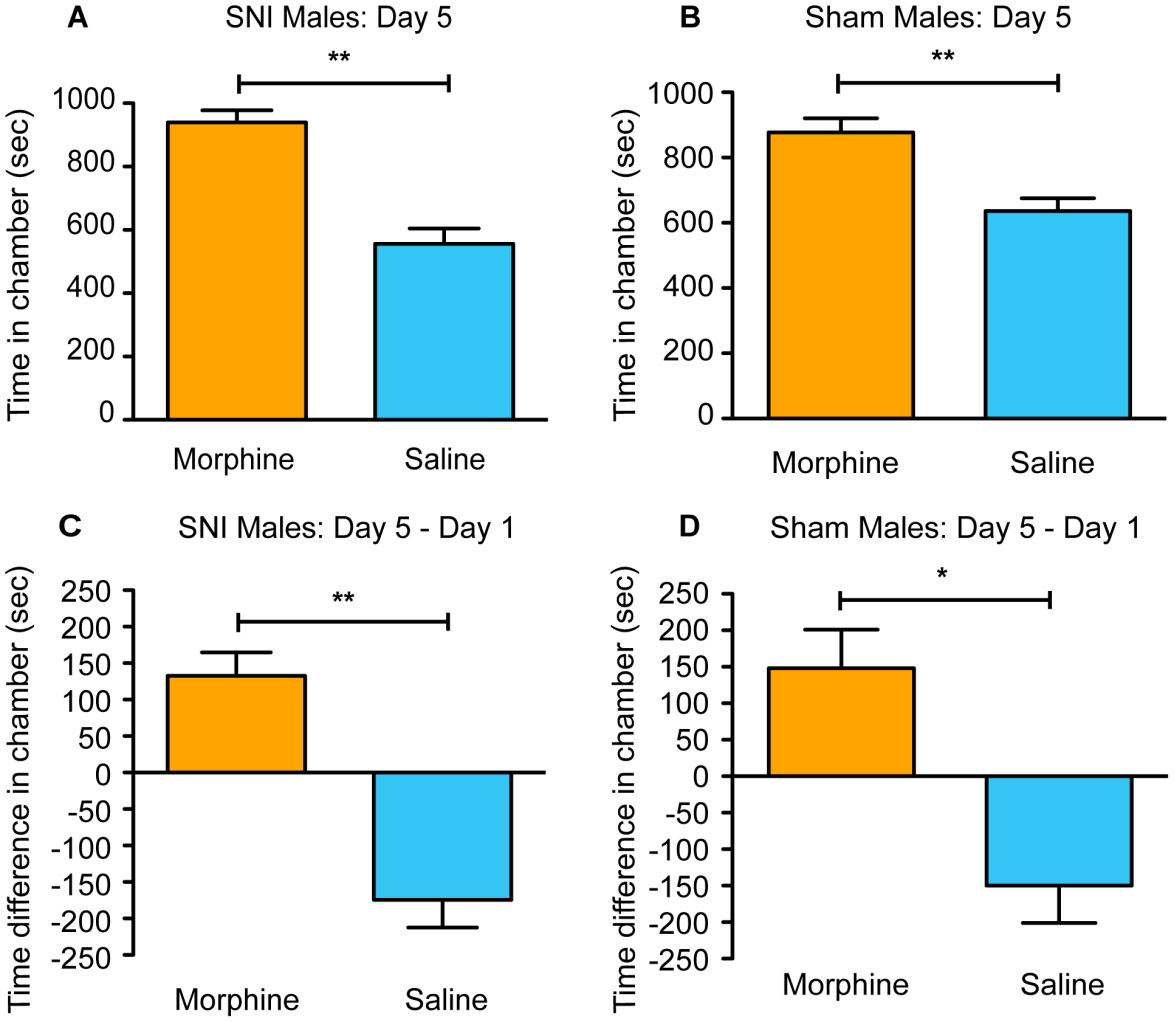
Morphine induces place preference in all male mice. (**A**) SNI mice (n = 9) spend significantly more total time in the morphine-paired chamber compared to vehicle-paired chamber on post-conditioning day 5. (**B**) Sham mice (n = 15) showed a similar significant preference. (**C**) SNI mice increase the amount of time they spent in the morphine-paired chamber on day 5 (post-conditioning) compared to baseline (day 1 pre-conditioning) while (**D**) sham mice showed a similar significant preference. Paired t-test, *p<0.05, **p<0.01.

We tested the effects of morphine in the aCPP assay in SNI and sham-operated mice. We looked at the total time spent in both the vehicle-paired chamber and the morphine-paired chamber on day 5, following three days of drug-pairing. Both SNI mice (n = 9) (Figure 5A; paired t-test, t_(8)_ = 4.56, p = 0.002) and sham mice (n = 15) (Figure 5B; paired t-test, t_(14)_ = 3.00, p = 0.009) spent more total time in the morphine-paired chamber than the vehicle-paired chamber on day 5. Next, we compared the time spent in each of the chambers on day 5 and day 1. Both SNI and sham mice spent more time in the morphine-paired chamber on day 5 (compared to day 1), while simultaneously decreasing the time spent in the saline-paired chamber (SNI Figure 5C, paired t-test, t_(8)_ = 4.63, p = 0.002; sham Figure 5D, paired t-test, t_(14)_ = 2.93, p = 0.011). Overall, when using morphine, preference was induced in both SNI and sham male mice.

### aCPP with morphine in female mice

**Figure 6:**
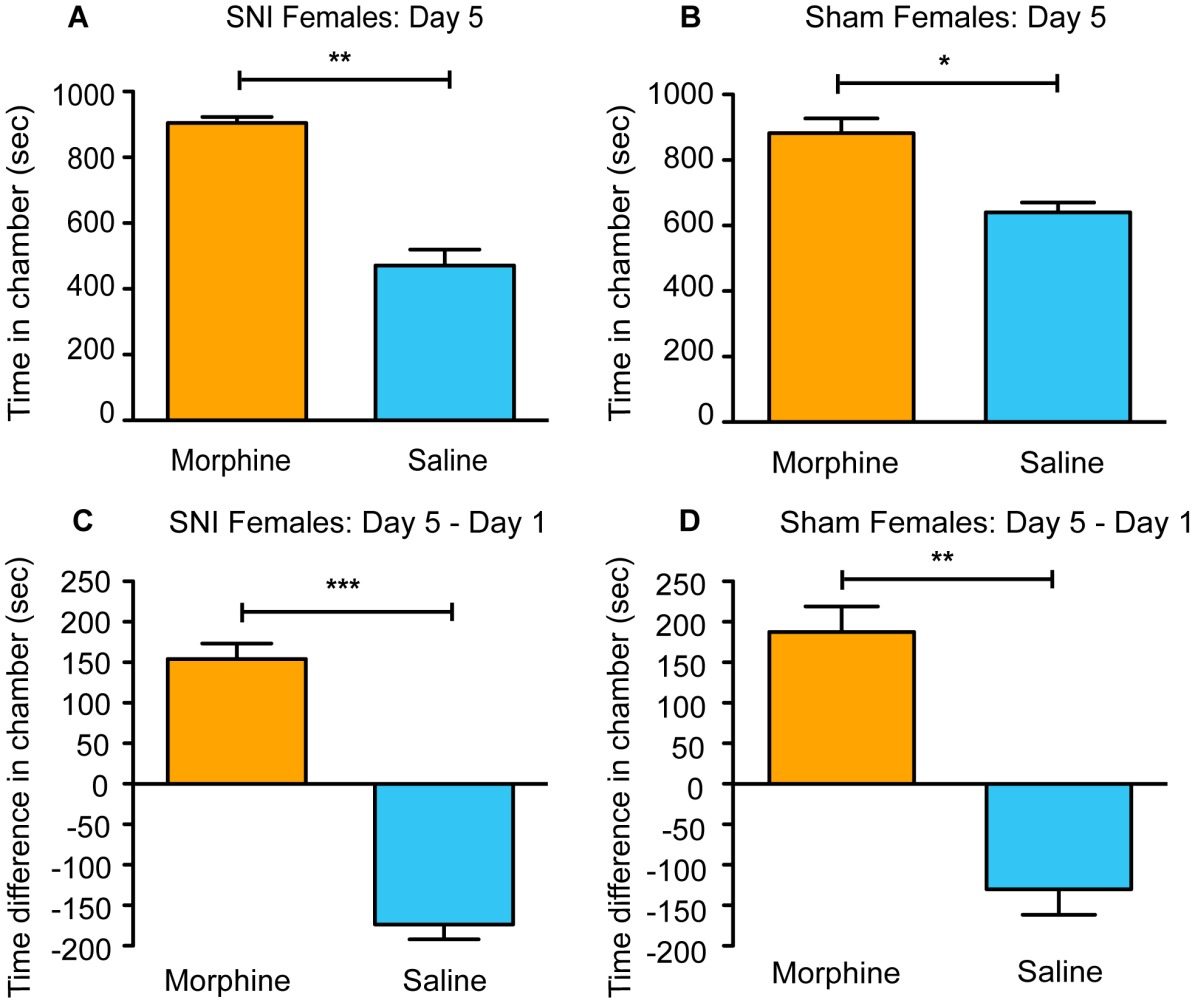
Morphine induces place preference in all female mice. (**A**) SNI mice (n = 5) spend significantly more total time in the morphine-paired chamber compared to vehicle-paired chamber on post-conditioning day 5. (**B**) Sham mice (n = 6) show a similar significant preference. (**C**) SNI mice increase the amount of time they spent in the morphine-paired chamber on day 5 (post-conditioning) compared to baseline (day 1 pre-conditioning). (**D**) Sham mice show a similar significant preference. Paired t-test, *p<0.05, **p<0.01, ***p<0.001.

Having demonstrated that morphine can induce conditioned place preference in male mice following three days of drug pairing, regardless of surgery treatment, next we tested the effect of morphine in female SNI and sham-operated mice. Both SNI (n = 5) (Figure 6A; paired t-test, t_(4)_ = 8.31, p = 0.001) and sham (n = 6) (Figure 6B; paired t-test, t_(5)_ = 3.72, p = 0.014) mice spent more total time in the morphine-paired chamber compared to the vehicle-paired chamber on day 5. We also compared the total amount of time spent in each chamber on day 5 compared to day 1 for both groups of mice. Again, similar to male mice, both SNI and sham female mice exhibited an increase in the time spent in the morphine-paired chamber on day 5 compared to day 1, along with a significant decrease in the time spent in the vehicle-paired chamber (SNI Figure 6C, paired t-test, t_(4)_ = 9.44, p = 0.001; sham Figure 6D, paired t-test, t_(5)_ = 6.49, p = 0.001). Overall, these data mirrored the results seen in males, with all mice spending more time in the side of the aCPP apparatus paired with morphine, regardless of surgery treatment.

## Discussion

mGluR5 has recently emerged as a potentially important target in pre-clinical reflexive and experimenter-induced pain assays and also may be a target for the development of new analgesic drugs. However, to date, there have been no studies that have looked at the benefit of fenobam or any other mGluR5 antagonist in models of on-going chronic pain-like behavior. Here, for the first time, we show that mGluR5 inhibition induces conditioned preference in animals with a chronic neuropathic injury. Furthermore, we show that both fenobam and MPEP do not induce statistically significant preference in sham mice. These data indicate that in the absence of pain-like injury, inhibition of mGluR5 is unlikely to have reinforcing properties on its own, a desirable quality for potential therapeutics. The injury-specific effects of fenobam and MPEP are in contrast to the non-specific effects of morphine seen in our studies. Morphine induces conditioned place preference in all mice regardless of injury status.

Fenobam was first developed as an anxiolytic drug by McNeil Laboratories [11]. Initially, it was shown to be effective in animal models of anxiety [29]. In human clinical trials, it was nearly as effective as the anxiolytic diazepam [29]. Fenobam eventually entered Phase II outpatient trials, but additional development ceased due to psychostimulant side-effects present at high doses (300 to 600 mg per patient) of the drug [30]. More recently, fenobam was found to act as an antagonist of mGluR5 [11] and interest greatly increased in this compound due to the fact that it is more selective for mGluR5 and has less off-target effects compared to MPEP [12]. Data from the last 20 years suggests that mGluR5 plays an important role in the regulation of pain [31]. Expression and activity of the receptor is implicated at numerous levels of the pain neuroaxis [32], including the periphery [33], the spinal cord [34] and the amygdala [35], [36]. Following the administration of intraperitoneally-delivered fenobam, the drug rapidly moves into the brain and is metabolized within one hour in mice [12]. While the rapid break down of fenobam is not ideal from a pharmacokinetic perspective, modification to its chemical structure could potentially lead to slower metabolism and extended effects. Recently, it was shown that the mGluR5 antagonist AZD9272 did not reduce pain in healthy male controls [37]. While this result is not supportive of mGluR5 in the modulation of control models of human pain, it does not necessarily indicate that all mGluR5 antagonists would be ineffective. AZD9272 is known to act centrally [37]; in our studies, fenobam may be acting both in the periphery and in the central nervous system, but this is unknown at this time. Future studies with peripherally restricted antagonists would be of interest to limit any potential for central side effects.

In a number of classic pain assays, both fenobam and MPEP have proven to reduce pain-like behaviors [12], [38]. However, classic inflammatory reflexive or experimenter-evoked (e.g. mechanical von Frey) models of persistent or acute pain to test novel analgesic compounds may be problematic. In particular, the predictive nature of these assays for efficacy in human clinical trials has been called into question [39]. Recently, a number of groups have validated a modified version of the conditioned place preference, aCPP, in the *post hoc* analysis of known human analgesic agents [21], [22], [40]. aCPP is an important new variation on the classic conditioned place preference that has been used for over 30 years to study drugs of abuse [20]. In the aCPP model, animals receive a nociceptive injury prior to testing. Next, pairing with known or unknown analgesic agents occurs. Animals develop a preference for the drug-paired chamber only if they receive relief from on-going or spontaneous nociception or other negative stimulation (e.g. anxiety). Other groups have shown that both mGluR1 [41] and mGluR5 [42], [43] antagonism inhibit the development of classic CPP to stimulant or rewarding drugs in naïve rats [41], [42] and mice [43]. Here, we show for the first time that systemic mGluR5 inhibition with fenobam or MPEP induces aCPP only in the context of a neuropathic injury, SNI. Both male and female SNI-operated mice developed a preference for the chamber paired with the mGluR5 antagonist. SNI mice spend a greater amount of time in the mGluR5 antagonist-paired chamber, compared to the vehicle chamber on day 5. In addition to this, there is also a significant increase in the time spent in the mGluR5 antagonist chamber on day 5 (post-conditioning) compared to the baseline day 1 (pre-conditioning). In contrast, male and female sham-operated mice show no statistically significant difference in the amount of time spent in the mGluR5 antagonist-paired chamber, when compared to the vehicle-paired chamber. These data suggest that mGluR5 antagonism is rewarding only in the context of SNI and that the rewarding effect may be caused by analgesia. The failure of both fenobam and MPEP to induce statistically significant preference in sham animals indicates that these drugs have a low potential for rewarding or deleterious effects in the absence of injury. In addition, this also highlights the value of aCPP as a screening paradigm. Avoiding agents that induce preference in naïve or sham-operated mice may reduce the chance of addiction in humans.

One additional aspect addressed in our studies is the fact that fenobam was able to produce preference in both male and female mice. This is important because there are many differences in pain condition prevalence between men and women [44]. For example, fibromyalgia [45] and interstitial cystitis [46] have been found to be much more prevalent in women compared to men. Despite this, many basic science studies only test male animals, neglecting the potential differences that could be found with females [47]. It is important to include both groups in studies in order to determine if the effects can be replicated in both men and women. Here, we were able to show that there were no sex differences between males and females regarding both fenobam and morphine aCPP. This suggests that mGluR5 antagonism with fenobam may be an effective strategy to broadly treat pain in both sexes.

Although the aCPP model represents an important step forward as a pain assay, it does not distinguish between analgesic effects of a drug versus affective effects. For example, chronic pain is often associated with comorbid depression and anxiety [48] and treatment with psychotherapy (pharmacological or non-pharmacological) can be beneficial to some patients. Mice [10], [49] and rats [39], [50] exhibit some comorbid depression/anxiety-like behavior with SNI. Thus, from the aCPP assay, it is not entirely clear whether mGluR5 antagonism induces place preference because it is reducing on-going pain or anxiety associated with SNI. Although we cannot differentiate between these two hypotheses, from a clinical standpoint, it may be sufficient to get relief from any negative stimulation, whether that stimulus is primarily nociceptive or primarily anxiogenic stimulation.

As described above, human clinical trials for fenobam were stopped in part because of observed psychostimulant effects at a dose that is much higher (300–600 mg per patient per day) than what we used in the present study [29]. In addition, mice exhibit hyperlocomotion to acute treatment of 30 mg/kg fenobam [13]. These data suggest that fenobam, and mGluR5 antagonists in general, may have unwanted stimulant effects that may or may not be related to analgesic efficacy. In the present study, we find that the stimulatory and rewarding effects of fenobam can be disassociated and that the stimulatory effects may be short term. In the first two days of pairing, SNI and sham mice injected with fenobam traveled more than animals injected with vehicle. These data show that all mice, irrespective of neuropathic injury, respond to the stimulatory effects of fenobam. This is particularly interesting because on post-conditioning day 5, the SNI mice only prefer to spend significantly more time in the fenobam-paired chamber. This time preference in SNI mice demonstrates that the rewarding effects of the drug in the context of injury are separate from its locomotor effects. Of note, the locomotor effects of fenobam are not seen on the last day of pairing, day 4. It is unclear whether this lack of an effect is indicative of habituation to fenobam-induced hyperactivity and/or a phenomenon associated with repeated testing habituation to the environment.

DMSO was chosen as the vehicle for fenobam because of fenobam's limited solubility in other solvents and the established use of DMSO in studies showing the analgesic effects of fenobam [12], [13], [26]. Furthermore, the pharmacokinetics of fenobam, which are important for demonstrating aCPP, were established in mice with a DMSO vehicle [13]. Chronic administration of DMSO for 14 days has not been shown to cause any liver or metabolic abnormalities nor any changes in sensory/motor behavior in mice [13]. The analgesic effects of fenobam are maintained with chronic pre-dosing with fenobam or DMSO, suggesting that in this context DMSO is not masking or altering the behavioral effects of fenobam. Furthermore, since we were able to replicate the fenobam results with a different mGluR5 antagonist (MPEP) that is soluble in saline, we are confident that the effects we observed are due to the antagonism of mGluR5 and not a confounding effect of DMSO. Nonetheless, due to the potential for side-effects of DMSO as a vehicle, future studies with fenobam may explore alternative vehicle options. Of note, fenobam does show good oral bioavailability in powdered form for human studies [37].

Finally, our results with the morphine aCPP show the benefit of aCPP as a screening tool for addictive potential of analgesic compounds, allowing the researcher to separate euphoria-inducing drugs (e.g. morphine) from non-euphoric drugs (e.g. fenobam and MPEP). Morphine preference has been demonstrated in naïve rats and mice, as well as in mice with spinal cord injury (SCI) [40], [51], [52]. As far as we know, this is the first report of the behavioral effects of morphine in SNI mice. Not surprisingly, all male and female animals, regardless of injury, show preference for morphine. It is likely that there is an interaction between the analgesic and euphoric effects of morphine. Our dose of morphine (10 mg/kg) has been shown to be analgesic [53] and has also been shown to induce preference in naïve mice in the classic conditioned place preference assay [27], [28]. This preference is likely developing in the sham animals through a euphoria-like mechanism and in the SNI mice through both analgesic and euphoria-induced positive reinforcement. Overall, data from our morphine experiments help demonstrate the significant contrast of mGluR5 antagonists compared to mu opioid receptor agonists like morphine. We show that mGluR5 inhibition with fenobam or MPEP does not induce preference in sham animals at a dose that is analgesic [12] in injured mice.

Overall, mice with induced neuropathic injury develop a preference for fenobam or MPEP after three days of pairing in the aCPP assay. Mice without SNI show no such preference. Since fenobam and MPEP do not show any deleterious effects in these sham mice, these data suggest that these drugs have no positive reinforcing effect in the absence of pain and induce preference only when a chronic injury is present. Finally, the locomotor side effects of fenobam are distinct from the analgesic effects of the drug. Our results demonstrate that fenobam and more broadly mGluR5 antagonists may have a promising future in the treatment of chronic pain.
